# Support vector data description with kernel density estimation (SVDD-KDE) control chart for network intrusion monitoring

**DOI:** 10.1038/s41598-023-46719-3

**Published:** 2023-11-06

**Authors:** Muhammad Ahsan, Hidayatul Khusna, Wibawati, Muhammad Hisyam Lee

**Affiliations:** 1 Department of Statistics, Institut Teknologi Sepuluh Nopember, Surabaya, Indonesia; 2 Department of Mathematical Sciences, Universiti Teknologi Malaysia, Johor Bahru, Malaysia

**Keywords:** Statistics, Information technology

## Abstract

Multivariate control charts have been applied in many sectors. One of the sectors that employ this method is network intrusion detection. However, the issue arises when the conventional control chart faces difficulty monitoring the network-traffic data that do not follow a normal distribution as required. Consequently, more false alarms will be found when inspecting network traffic data. To settle this problem, support vector data description (SVDD) is suggested. The control chart based on the SVDD distance can be applied for the non-normal distribution, even the unknown distributions. Kernel density estimation (KDE) is the nonparametric approach that can be applied in estimating the control limit of the non-parametric control charts. Based on these facts, a multivariate chart based on the integrated SVDD and KDE (SVDD-KDE) is proposed to monitor the network's anomaly. Simulation using the synthetic dataset is performed to examine the performance of the SVDD-KDE chart in detecting multivariate data shifts and outliers. Based on the simulation results, the proposed method produces better performance in detecting shifts and higher accuracy in detecting outliers. Further, the proposed method is applied in the intrusion detection system (IDS) to monitor network attacks. The NSL-KDD data is analyzed as the benchmark dataset. A comparison between the SVDD-KDE chart with the other IDS-based-control chart and the machine learning algorithms is executed. Although the it has high computational cost, the results show that the IDS based on the SVDD-KDE chart produces a high accuracy at 0.917 and AUC at 0.915 with a low false positive rate compared to several algorithms.

## Introduction

Network, computers, and technology play a significant part in daily life. However, network attacks have disturbed their merits in recent years. The intrusion detection system (IDS) is a functional security component that inspects the network connections and prevents suspicious packages^1^. Many studies related to intrusion detection have been carried out using machine learning methods. Several algorithms of machine learning have been applied in IDS, such as naïve Bayes (NB)^2,3^, logistic regression (LR)^4,5^, decision tree (DT)^6^, random forest (RF)^7–9^, and support vector machine (SVM)^3,4^, support vector data description (SVDD)^10,11^, convolutional neural network (CNN)^12,13^, recurrent neural network (RNN)^14,15^, and long-short-term memory (LSTM)^16,17^.

Intrusion detection can be conducted by scanning anomalies or suspicious network traffic patterns^18^. These network anomalies can be analogized as out-of-control samples or outliers in monitoring quality using a control chart. Hence, the statistical process control (SPC) method, especially the multivariate chart, can be utilized in IDS^19^. The utilization of the IDS-based multivariate control chart in inspecting the network traffic anomalies can be a powerful tool to protect the safety and reliability of the network^20^.

Several types of research have been performed in applying multivariate control charts in IDS. Abdel-Aziz et al.^21^ used the multivariate chart for network anomaly monitoring. The combination of the *T*^2^ chart with successive difference covariance matrix (SDCM) for IDS shows acceptable results for finding network attacks^22^. IDS-based Robust Hotelling's *T*^2^ chart using the adaptive control limit with kernel density estimation (KDE) displayed a faster computational time without lowering accuracy and precision^23^. The PCA-based *T*^2^ chart using the robust estimator fast minimum covariance determinant (FMCD) and KDE control limit a lower False Negative and higher accuracy than the other charts^24^. The PCA Mix and Kernel PCA (KPCA) Mix control chart perform better in detecting network anomalies than the other methods^25,26^.

Although it has been widely used, there are some issues with the IDS-based multivariate control chart. Majority of the multivariate charts are developed under a certain distribution as stated by Ahsan et al.^27^. Zhu highlighted that the network traffics hard to have the multivariate normal distribution caused by extreme values from the intrusions^28^. As a consequence, there will be many false alarms occur.

Furthermore, most of the multivariate control charts used in IDS are Hotelling’s *T*^2^. However, the statistic of *T*^2^ can be easily affected by the outliers^29^. As a result, its ability to detect anomalies can be decreased^30^. These conditions threaten the security and stability of the system because the system has a lower detection rate and produces more false alarms^23^.

To overcome this situation, the support vector data description (SVDD) algorithms can be applied to increase the detection rate and solve the problem of non-normality. SVDD is a single-category label developed based on the SVM method to detect outliers. This method was originally proposed by^31^. The SVDD-based control chart can be used when the distributions of quality characteristics are relatively varied or even unknown. Using kernel functions in SVDD can create boundaries that follow the normal data connection data pattern without having to follow a certain distribution. By using this method, more anomalies or attacks can be identified.

Furthermore, the utilization of KDE can be an alternative to solve the high false alarm issue. This method can create the control limit by using the information of the normal connection data. Some IDS have been employing this method^23,24,32^. The capability of KDE method in estimating the empirical distribution from various types of data patterns, decreasing false positives rate or swamping effect of the IDS proposed.

Based on the problems above, this research suggests the combination of multivariate chart based on SVDD and KDE (SVDD-KDE) to inspect the anomalies in the network. First, the SVDD-KDE chart’s performance is assessed to detect process shifts using the average run length (ARL) criterion. Further, the performance of the proposed SVDD-KDE chart is also examined to detect the outlier. Finally, the proposed SVDD-KDE chart is employed to observe the synthetic dataset and network traffic. The NSL-KDD dataset is used as the benchmark of the IDS. Also, the performance of the proposed IDS based on the SVDD-KDE chart is compared to several control charts and machine learning algorithms.

The remains of this paper are composed as follows: The procedures of the proposed SVDD-KDE chart are elaborated in Section “Proposed SVDD-KDE chart”. The performance of the proposed SVDD-KDE chart is provided in Section “SVDD-KDE control chart performance”. Section “IDS-based proposed control chart algorithm” discusses the proposed IDS algorithms. The utilization of the proposed IDS based on the SVDD-KDE chart in detecting network anomalies is discussed in Section “Application for monitoring network anomaly”. In the end, Section “Conclusions” is assigned for the conclusions and suggestions for future research.

## Proposed SVDD-KDE chart

### Support vector data description (SVDD)

Let ${\mathbf{x}}_{i} = [x_{i1} ,x_{i2} ,...,x_{ip} ]{\prime} ,\,\,{\text{where}}\,\,i = 1,2,...,n,$ be a column vector with dimension *p*, where **x**_*i*_ are the training data. To fit the sphere around the target data, the sphere is determined by the quadratic programming solution as follows:

1 $$ {\text{Minimize}}\;\;F(R,{\mathbf{a}}) = R^{2} + \kappa \sum\nolimits_{i} {\varsigma_{i} } $$

subject to

2 $$ \left\| {{\mathbf{x}}_{i} - {\mathbf{a}}} \right\|^{2} \le R^{2} + \varsigma_{i} ,\;\;\;\varsigma_{i} \ge 0, $$

where *F*, **a,** and *R* are the cost functions for minimizing the center and sphere radius, respectively. The slack variable that allows the outlier detection in the training data is symbolized as $\varsigma_{i}$. If $\kappa > 0$ is a penalty parameter which supervises the change from volume sphere and misclassification, Eq. (2) can be substituted into Eq. (1) with the Lagrange multipliers as follows:

3 $$ L(R,{\mathbf{a}},{{\varvec{\upalpha}}},{{\varvec{\upgamma}}},{\mathbf{\varsigma }}) = R^{2} + \kappa \sum\limits_{i = 1}^{n} {\varsigma_{i} - } \sum\limits_{i = 1}^{n} {\alpha_{i}^{*} \left[ {R^{2} - \varsigma_{i} - \left\| {{\mathbf{x}}_{i} - {\mathbf{a}}} \right\|^{2} } \right] - \sum\limits_{i = 1}^{n} {\gamma_{i} \varsigma_{i} } } $$

where ${\alpha }_{i}^{*}\ge 0$ and ${\gamma }_{i}\ge 0$. The dual problem in Eq. (2) is rewritten into the following equation:

4 $$ Max\,\,L = \sum\limits_{i = 1}^{n} {\alpha_{i}^{*} \left( {{\mathbf{x}}_{i} \cdot {\mathbf{x}}_{j} } \right)} $$

subject to

5 $$ \sum\limits_{i = 1}^{n} {\alpha_{i}^{*} = 1} , $$

where $0 \le \alpha_{i}^{*} \le \kappa ,$. The distance among the support vectors and the hypersphere center is called the hypersphere radius and is formulated as follows:

6 $$ R^{2} = \left\| {{\mathbf{x}}_{k} - {\mathbf{a}}} \right\|^{2} = \left( {{\mathbf{x}}_{k} \cdot {\mathbf{x}}_{k} } \right) - 2\sum\limits_{i = 1}^{n} {\alpha_{i}^{*} K\left( {{\mathbf{x}}_{i} \cdot {\mathbf{x}}_{k} } \right)} + \sum\limits_{i,j = 1}^{n} {\alpha_{i}^{*} \alpha_{j}^{*} \left( {{\mathbf{x}}_{i} \cdot {\mathbf{x}}_{k} } \right)} , $$

where ${\mathbf{x}}_{k}$ are the support vectors. Furthermore, the distance from the test data ***z*** to the center of the hypersphere needs to be calculated. The inner product $\left( {{\mathbf{x}}_{i} \cdot {\mathbf{x}}_{j} } \right)$ in Eqs. (4) and (6) can be replaced with a kernel function to make the SVDD method more flexible for outlier detection. The formula for the calculation is defined:

7 $$ D^{2} = K\left( {{\mathbf{z}}^{\prime} \cdot {\mathbf{z}}} \right) - 2\sum\limits_{i = 1}^{n} {\alpha_{i}^{*} K\left( {{\mathbf{x}}_{i} \cdot {\mathbf{z}}} \right)} + \sum\limits_{i,j = 1}^{n} {\alpha_{i}^{*} \alpha_{j}^{*} \left( {{\mathbf{x}}_{i} \cdot {\mathbf{x}}_{k} } \right)} , $$

In this research, the kernel function applied in SVDD is the radial basis function (RBF) kernel and is expressed as:

8 $$ K\left( {{\mathbf{x}}_{i} \cdot {\mathbf{x}}_{j} } \right) = \exp \left[ { - \frac{{\left\| {{\mathbf{x}}_{i} \cdot {\mathbf{x}}_{j} } \right\|^{2} }}{w}} \right], $$

where *w* is the hyperparameter of RBF kernel. The distance $D^{2}$ is used as the statistics plotted on the proposed control chart, and its control limit is calculated using the KDE method.

### Kernel density estimation

The KDE can be used in estimating the empirical probability density function (pdf) from an unspecified distribution of random variables. Under the in-control state, the empirical *D*^2^ distribution can be estimated using KDE to compute its control limit. The kernel function is adopted in order to estimate the empirical distribution of the *D*^2^ statistic as follows:

9 $$ \hat{f}_{h} (d) = \frac{1}{n}\sum\limits_{i = 1}^{n} {K\left[ {\frac{{\left( {d - D_{i}^{2} } \right)}}{{\hat{\rho }}}} \right]} , $$

where $\hat{\rho }$ and *K* define the estimated smoothing parameter or bandwidth and the kernel function, respectively. To calculate the KDE control limit, the Gaussian Kernel is employed in this analysis. The control limit of SVDD-KDE control chart is estimated from ($100(1 - \alpha )$-th) percentile of *D*^2^ empirical distribution and is determined using the following expression:

10 $$ CL_{{{\text{kernel}}}} = \hat{F}_{h} (d)^{ - 1} \left( {1 - \alpha } \right), $$

where $\alpha$ is the false alarm rate.

## SVDD-KDE control chart performance

This section presents the proposed SVDD-KDE chart’s performance. Three kinds of evaluation are conducted: performance in detecting process shift, performance in detecting outlier, and performance in monitoring the synthetic dataset.

### Performance for detecting process shift

This subsection presents the performance evaluation of the proposed SVDD-KDE control chart in identifying process shifts. The simulation study is conducted to evaluate the performance of the proposed chart using the average run length (ARL) criterion. If the mean vector is written as ${{\varvec{\upmu}}}$ and covariance matrix is expressed as $\,{{\varvec{\Sigma}}}$, the data **X** are generated following the multivariate normal distribution with ${{\varvec{\upmu}}} = {\mathbf{0}}$ and $\,{{\varvec{\Sigma}}}{ = }{\mathbf{I}}$, or in other terms ${\mathbf{X}}\sim N_{p} \left( {{\mathbf{0}},{\mathbf{I}}} \right)$. When the process is in-control (shift ${{\varvec{\updelta}}} = {\mathbf{0}}$) the ARL_0_ is utilized to assess the performance of the SVDD-KDE chart. The target of ARL_0_ for this simulation study is 370 which refers to the 3-sigma rule. Furthermore, the ARL_1_ is calculated by increasing the mean vector for each variable characteristic ${{\varvec{\upmu}}}_{shift} = {{\varvec{\upmu}}} + {{\varvec{\updelta}}},$ where ${{\varvec{\updelta}}}_{\mu } = {\mathbf{0}}{\mathbf{.1}} = [0.1\,\,\,0.1\,\,...\,\,\,0.1]^{\prime}_{1 \times p}$. The SVDD-KDE chart’s performance is evaluated and is compared with Hotelling’s *T*^2^ chart.

Table 1 presents the performance comparison of the proposed SVDD-KDE chart with Hotelling’s *T*^2^ chart for *p* = 2, 3, 5, and 7. When there is no shift in the process, both charts produce a similar ARL_0_ $\approx \,370$(bold value in table). For the shifted process, the SVDD-KDE chart has a preferable performance to Hotelling’s *T*^2^ chart for a small and large shift which can be noticed from the lower value of ARL_1_. Also, it can be that performance of the SVDD-KDE chart gets better as the number of quality characteristics gets larger.

**Table 1:** Performance for Different Quality Characteristics.Significant values are in bold.

Shift (${{\varvec{\updelta}}}$)	*p* = 2		*p* = 3		*p* = 5		*p* = 7	
	Proposed chart	*T*^2^	Proposed chart	*T*^2^	Proposed chart	*T*^2^	Proposed chart	*T*^2^
0.0	**376.13**	**366.39**	**379.15**	**354.91**	**386.65**	**369.51**	**378.32**	**367.09**
0.2	271.95	299.69	256.65	297.06	239.65	296.08	217.61	291.58
0.4	151.35	187.80	155.45	176.08	148.65	140.72	128.35	136.53
0.6	108.15	98.07	66.50	81.75	46.52	62.96	33.36	53.42
0.8	48.55	50.70	31.57	37.96	29.25	27.34	21.65	20.75
1.0	20.43	27.34	14.42	20.85	24.35	13.04	18.05	9.26
1.2	12.32	15.83	12.12	10.92	10.25	6.88	9.65	4.56
1.4	8.77	10.08	5.85	6.64	5.05	3.68	4.15	2.60
1.6	4.83	6.49	3.65	4.20	3.45	2.42	3.12	1.73
1.8	3.32	4.26	2.05	2.77	1.43	1.71	1.35	1.32
2.0	2.51	3.21	1.78	2.14	1.25	1.33	1.15	1.14
2.2	2.15	2.32	1.75	1.63	1.22	1.19	1.05	1.04
2.4	1.65	1.85	1.13	1.34	1.21	1.06	1.01	1.01
2.6	1.45	1.52	1.15	1.16	1.05	1.02	1.00	1.01
2.8	1.24	1.28	1.05	1.10	1.00	1.01	1.00	1.00
3.0	1.00	1.22	1.00	1.05	1.00	1.00	1.00	1.00

Table 2 presents the SVDD-KDE chart's performance for several types of correlation. For the in-control process, it is visible that the proposed chart yields the stable ARL_0_ at about 370. For the shifted process, it can be noticed that the SVDD-KDE chart performs better for the higher correlation in detecting the small process shift. On, for the smaller correlation, the SVDD-KDE chart has a better performance in identifying the larger process shift.

**Table 2:** Performance of the proposed chart for different correlation.

Shift (${{\varvec{\updelta}}}$)	$\rho$					
	0	0.2	0.3	0.5	0.7	0.9
0.0	**379.15**	**376.93**	**383.6**	**374.5**	**385.35**	**372.15**
0.2	256.65	255.26	254.3	242.95	222.21	215.86
0.4	155.45	155.55	165.15	155.85	105.32	101.79
0.6	66.50	66.72	65.15	64.25	63.92	62.82
0.8	31.57	31.25	31.65	31.13	31.45	25.12
1.0	14.42	14.95	14.72	14.18	14.85	15.25
1.2	12.12	12.81	12.72	12.44	13.15	13.83
1.4	5.85	6.25	8.15	8.03	10.31	11.75
1.6	3.65	3.73	3.71	3.65	6.14	10.15
1.8	2.05	2.45	2.62	3.15	5.75	7.28
2.0	1.78	2.05	2.75	3.75	3.82	6.15
2.2	1.75	1.95	2.46	3.05	3.15	4.35
2.4	1.13	1.72	2.25	2.35	2.45	3.24
2.6	1.15	1.15	1.25	1.52	1.60	2.25
2.8	1.05	1.15	1.55	1.45	1.30	1.45
3.0	1.00	1.00	1.00	1.00	1.10	1.12

Significant values are in bold.

### Performance for detecting outlier

In this subsection, the proposed SVDD-KDE chart’s performance is appraised for the different kinds of outliers. The percentages of outliers $$\varepsilon$$ that are contaminated with the clean or normal data are 5%, 10%, 15%, 20%, 30%, and 50% over the number of observations. Similar to the previous subsection, the clean data $${\mathbf{X}}_{clean}$$ are generated following multivariate normal distribution with a $${{\varvec{\upmu}}}_{clean} = {\mathbf{0}}$$ and $$\,{{\varvec{\Sigma}}}{ = }{\mathbf{I}}$$, $${\mathbf{X}}_{clean} \sim N_{p} \left( {{{\varvec{\upmu}}}_{clean} ,{\mathbf{I}}} \right)$$. The experimental studies are done for different quality characteristics, such as *p* = 3, 5, 10, 15, 20, and 30. The contaminated data $${\mathbf{X}}_{cont}$$ are generated following the Multivariate Normal Distribution with $${{\varvec{\upmu}}}_{cont} = {\mathbf{3}}$$$$= [3\,\,\,3\,\,...\,\,\,3]^{\prime}_{1 \times p}$$ and $$\,{{\varvec{\Sigma}}}{ = }{\mathbf{I}},$$$${\mathbf{X}}_{cont} \sim N_{p} \left( {{{\varvec{\upmu}}}_{cont} ,{\mathbf{I}}} \right)$$.

Table 3 tabulated the confusion matrix for detecting outliers. The proposed SVDD-KDE chart’s performance in detecting outliers from the simulated data is assessed by 3 metrics as follows:

1. ${\text{Hit}}\,{\text{Rate}} = \frac{True\,Positive\,(TP) + True\,Negative\,(TN)}{n},\,$ where *n* is the number of observations.
2. $$Fasle\,Positive\,(FP)\,{\text{Rate}} = \frac{Fasle\,Positive\,(FP)}{{TrueNegative\,(TN) + Fasle\,Positive\,(FP)}}.$$
3. $$False\,Negative\,(FN)\,{\text{Rate}} = \frac{False\,Negative\,(FN)}{{True\,positive\,(TP) + False\,Negative\,(FN)}}.$$
4. $$Area \, under \, curve\left( {AUC} \right) = \frac{1}{2}\left( {\frac{TP}{{TP + FN}} + \frac{TN}{{TN + FP}}} \right)$$.

**Table 3:** Outlier detection confusion matrix.

Actual label	Estimated Label	
	Outlier	Normal
Outlier	TP	FN
Normal	FP	TN

In calculating the FN Rate, FP Rate, and Hit Rate the simulation is repeated 1000 times. Tables 4, 5, 6, 7, 8 and 9 present the performance comparison of Hotelling’s *T*^2^ and the SVDD-KDE chart for detecting outliers. From the simulation results, it can be seen that for the number of outliers contaminated with the clean data lower than 30%, the proposed chart shows better results (the lower FN rate with higher accuracy or Hit Rate). This indicates that the SVDD-KDE chart has a better detection rate than Hotelling’s *T*^2^ chart. For the larger number of outliers added to the clean or contaminated data (30% and 50%), the performance of both charts is relatively similar.

**Table 4:** Performance comparison in detecting outlier for $\varepsilon =$ 5%.

Number of quality characteristics	Hit rate		AUC		FP rate		FN rate	
	Proposed chart	*T*^2^	Proposed chart	*T*^2^	Proposed chart	*T*^2^	Proposed chart	*T*^2^
*p* = 3	0.9943	0.9685	0.9600	0.6951	0.0017	0.0011	0.0784	0.6088
*p* = 5	0.9973	0.9672	0.9971	0.6853	0.0027	0.0015	0.0032	0.6280
*p* = 10	0.9975	0.9620	0.9987	0.6363	0.0027	0.0018	0.0000	0.7256
*p* = 15	0.9973	0.9576	0.9986	0.5906	0.0028	0.0016	0.0000	0.8172
*p* = 20	0.9963	0.9562	0.9982	0.5767	0.0037	0.0016	0.0000	0.8450
*p* = 30	0.9945	0.9536	0.9973	0.5541	0.0055	0.0020	0.0000	0.8898

**Table 5:** Performance comparison in detecting outlier for $\varepsilon =$ 10%.

Number of quality characteristics	Hit rate		AUC		FP rate		FN rate	
	Proposed chart	*T*^2^	Proposed chart	*T*^2^	Proposed chart	*T*^2^	Proposed chart	*T*^2^
*p* = 3	0.9892	0.9044	0.9516	0.5250	0.0012	0.0008	0.0956	0.9492
*p* = 5	0.9976	0.9041	0.9964	0.5272	0.0021	0.0016	0.0051	0.9441
*p* = 10	0.9985	0.9021	0.9991	0.5163	0.0018	0.0015	0.0000	0.9660
*p* = 15	0.9979	0.9011	0.9989	0.5113	0.0023	0.0015	0.0000	0.9759
*p* = 20	0.9977	0.9006	0.9988	0.5098	0.0025	0.0017	0.0000	0.9787
*p* = 30	0.9961	0.8999	0.9978	0.5072	0.0045	0.0019	0.0000	0.9837

**Table 6:** Performance comparison in detecting outlier for $\varepsilon =$ 15%.

Number of quality characteristics	Hit rate		AUC		FP rate		FN rate	
	Proposed chart	*T*^2^	Proposed chart	*T*^2^	Proposed chart	*T*^2^	Proposed chart	*T*^2^
*p* = 3	0.9802	0.8518	0.9352	0.5075	0.0003	0.0006	0.1294	0.9845
*p* = 5	0.9981	0.8515	0.9972	0.5087	0.0015	0.0015	0.0041	0.9811
*p* = 10	0.9990	0.8502	0.9995	0.5049	0.0011	0.0017	0.0000	0.9886
*p* = 15	0.9987	0.8500	0.9993	0.5032	0.0015	0.0014	0.0000	0.9922
*p* = 20	0.9985	0.8498	0.9992	0.5038	0.0017	0.0019	0.0000	0.9905
*p* = 30	0.9972	0.8497	0.9984	0.5025	0.0033	0.0015	0.0000	0.9935

**Table 7:** Performance comparison in detecting outlier for $\varepsilon =$ 20%.

Number of quality characteristics	Hit rate		AUC		FP rate		FN rate	
	Proposed chart	*T*^2^	Proposed chart	*T*^2^	Proposed chart	*T*^2^	Proposed chart	*T*^2^
*p* = 3	0.9556	0.8007	0.8899	0.5040	0.0003	0.0015	0.2200	0.9906
*p* = 5	0.9974	0.8003	0.9950	0.5024	0.0009	0.0012	0.0092	0.9941
*p* = 10	0.9995	0.7999	0.9996	0.5022	0.0008	0.0016	0.0000	0.9941
*p* = 15	0.9910	0.8000	0.9995	0.5021	0.0011	0.0013	0.0000	0.9946
*p* = 20	0.9984	0.7998	0.9992	0.5018	0.0017	0.0015	0.0000	0.9950
*p* = 30	0.9982	0.7995	0.9989	0.5016	0.0022	0.0019	0.0000	0.9949

**Table 8:** Performance comparison in detecting outlier for $\varepsilon =$ 30%.

Number of quality characteristics	Hit rate		AUC		FP rate		FN rate	
	Proposed chart	*T*^2^	Proposed chart	*T*^2^	Proposed chart	*T*^2^	Proposed chart	*T*^2^
*p* = 3	0.7003	0.7003	0.5014	0.5011	0.0018	0.0009	0.9954	0.9969
*p* = 5	0.7002	0.7000	0.5019	0.5012	0.0031	0.0017	0.9931	0.9960
*p* = 10	0.7000	0.6997	0.5016	0.5007	0.0023	0.0016	0.9945	0.9971
*p* = 15	0.6941	0.6999	0.5165	0.5009	0.0028	0.0015	0.9642	0.9968
*p* = 20	0.6994	0.6996	0.5010	0.5007	0.0038	0.0020	0.9942	0.9966
*p* = 30	0.6991	0.6995	0.5015	0.5006	0.0046	0.0022	0.9924	0.9967

**Table 9:** Performance comparison in detecting outlier for $\varepsilon =$ 50%

Number of quality characteristics	Hit rate		AUC		FP rate		FN rate	
	Proposed chart	*T*^2^	Proposed chart	*T*^2^	Proposed chart	*T*^2^	Proposed chart	*T*^2^
*p* = 3	0.4999	0.5000	0.4998	0.5000	0.0022	0.0010	0.9982	0.9990
*p* = 5	0.5000	0.4999	0.5000	0.5000	0.0037	0.0020	0.9963	0.9981
*p* = 10	0.5001	0.4999	0.5005	0.4999	0.0024	0.0019	0.9966	0.9983
*p* = 15	0.4996	0.5000	0.5003	0.5001	0.0240	0.0021	0.9755	0.9978
*p* = 20	0.4997	0.5000	0.5004	0.5000	0.0038	0.0026	0.9954	0.9974
*p* = 30	0.5000	0.5000	0.4998	0.5000	0.0059	0.0023	0.9946	0.9977

### Performance for simulated data

This subsection displays the proposed SVDD-KDE chart’s ability to monitor the simulated data. The 100 simulated data is generated to follow multivariate normal distribution with correlation $\rho$ = 0.3 with *p* = 3. The first 70 data is generated with ${{\varvec{\upmu}}} = {\mathbf{0}}$, while the remaining 30 data is generated with shifted mean vector ${{\varvec{\upmu}}}_{shift} = {{\varvec{\upmu}}} + {{\varvec{\upsigma}}}$ The performance is evaluated for the small shift (${{\varvec{\updelta}}} = {\mathbf{0}}{\mathbf{.5}}$), moderate shift (${{\varvec{\updelta}}} = {\mathbf{1}}{\mathbf{.5}}$), and large shift (${{\varvec{\updelta}}} = {\mathbf{3}}$). To find the best hyperparameter for the studied case, several values of hyperparameter are used, as presented in Table 10.

**Table 10:** Scenarios of Simulated data.

Scenarios	Number of quality characteristics	$\rho$	*w*
S1	3	0.3	2.5
S2	3	0.3	1.5
S3	3	0.3	1.0
S4	3	0.3	0.7
S5	3	0.3	0.5
S6	3	0.3	0.1

**Figure 1:**
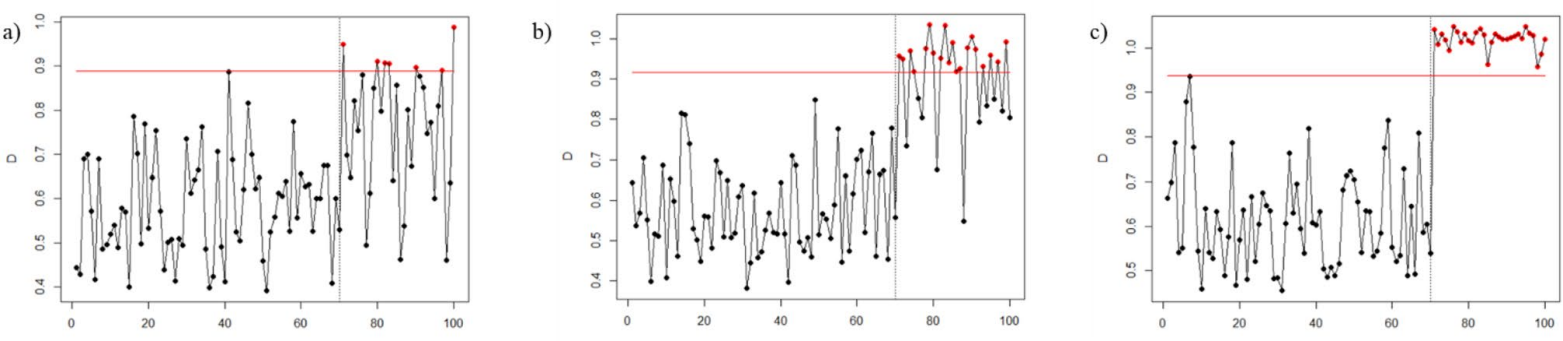
Performance of the SVDD-KDE chart in monitoring simulated data in the S1 for: (**a**) Small shift, (**b**) Moderate shift, and (**c**) Large shift.

**Figure 2:**
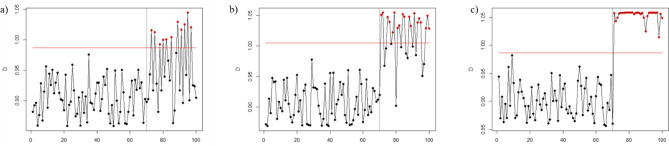
Performance of the SVDD-KDE chart in monitoring simulated data in S2 for: (**a**) Small shift, (**b**) Moderate shift, and (**c**) Large shift.

**Figure 3:**
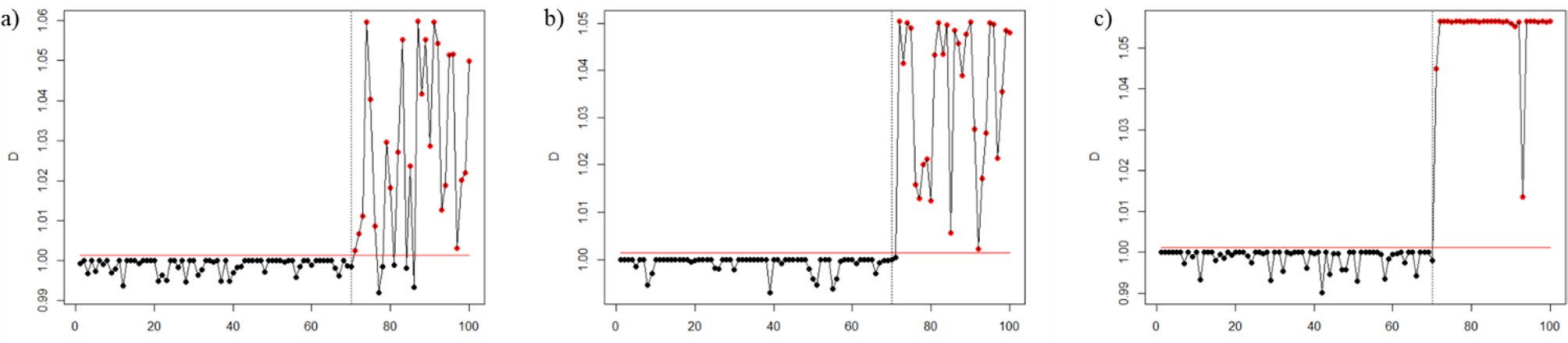
Performance of the SVDD-KDE chart in monitoring simulated data in S3 for: (**a**) Small shift, (**b**) Moderate shift, and (**c**) Large shift.

**Figure 4:**
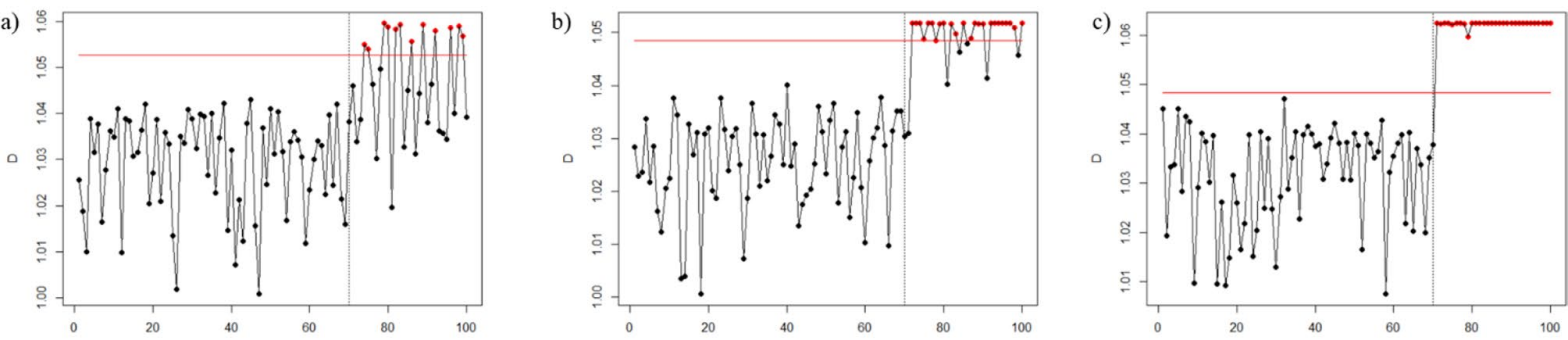
Performance of the SVDD-KDE chart in monitoring simulated data in S4 for: (**a**) Small shift, (**b**) Moderate shift, and (**c**) Large shift.

**Figure 5:**
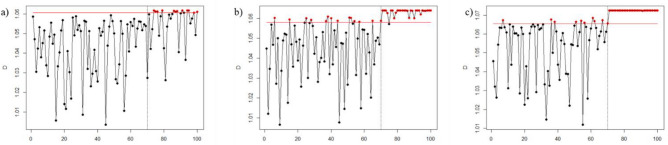
Performance of the SVDD-KDE chart in monitoring simulated data in S5 for: (**a**) Small shift, (**b**) Moderate shift, and (**c**) Large shift.

**Figure 6:**
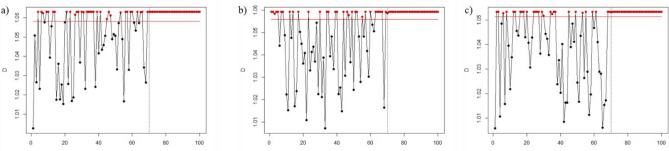
Performance of the SVDD-KDE chart in monitoring simulated data in S6 for: (**a**) Small shift, (**b**) Moderate shift, and (**c**) Large shift.

Figures 1, 2, 3, 4, 5, and 6 depict the proposed SVDD-KDE chart’s performance in inspecting the simulated data for small, moderate, and large shifts. In general, the proposed chart demonstrates better results for scenarios S1–S4, especially for the large shift. Based on the inspecting results, it is noticeable that scenario S3 (hyperparameter *w* = 1) produces better results. For scenarios S5 and S6, the performance of the SVDD-KDE chart is getting worse by producing more false alarms.

### Ethics approval

This work does not involve experiments on animals and humans.

## IDS-based proposed control chart algorithm

### IDS algorithm

**Figure 7:**
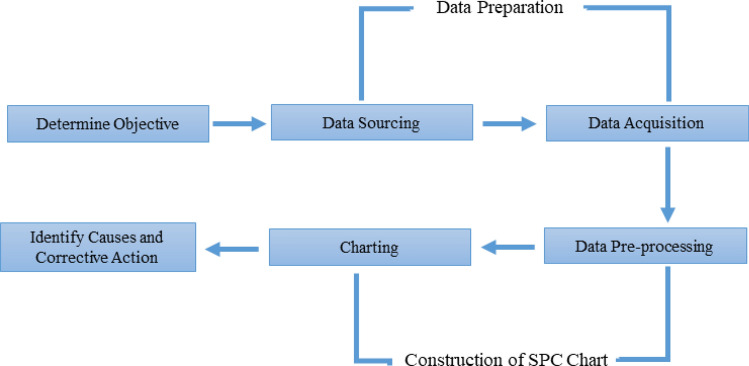
Intrusion detection system using a control chart method^19^.

The IDS based on the SVDD-KDE chart’s algorithms is explained in this section. Figure 7 depicts the monitoring anomalies procedures with control chart approach. In general, there are four main steps in this procedure: determining the objective, data preparation, control chart construction, identifying problems, and performing corrections for system improvement. Furthermore, the algorithms of the IDS based on the SVDD-KDE chart are split into 2 phases as follows:

#### Phase I: training phase

This phase includes the training process for the normal connection data. This is conducted to create a normal profile and estimate the KDE control limit. The estimated values of the hyperparameter are then used in the detection phase in testing and monitoring the new connections. The procedures of the training phase are assigned as follows:

**Step 1**: Specify hyperparameter of RBF Kernel *w* and false alarm rate $\alpha$.

**Step 2**: Create a matrix ${\mathbf{X}}_{normal}$, which contains the normal connection data.

**Step 3**: Calculate statistics *D*^2^ from normal labeled data ${\mathbf{X}}_{normal}$ using Eq. (7).

**Step 4**: Estimate the KDE control limit using $CL_{Kernel}$ from Eq. (10).

#### Phase II: testing and detection phase

The estimated hyperparameter values, mean of the in-control *D*^2^, and $CL_{Kernel}$ from training in Phase I are used in this phase. The procedures of the detection phase are defined as follows:

**Step 1**: Create a matrix ${\mathbf{X}}_{test}$, which is the new connection data.

**Step 2**: Calculate statistics *D*^2^ by testing new connection data using the hyperparameter from phase I.

**Step 3**: If $D_{i}^{2} > CL_{Kernel}$ then the connection is an intrusion and if $D_{i}^{2} < CL_{Kernel}$ then the connection is normal for $i = 1,2,...,n$.

## Application for monitoring network anomaly

### NSL-KDD dataset

This subsection presents a summary of the dataset used in this research. The NSL-KDD data is exploited in this paper to reveal the performance of the proposed SVDD-KDE chart in observing the network connection data. Table 11 gives the summary of the NSL-KDD dataset.

**Table 11:** Summary of NSL-KDD dataset.

Label	Number of connections	Label percentage
Normal	67,343	53.458
Attack	58,630	46.542
DOS	45,927	36.458
Probe	11,656	9.253
U2R	52	0.041
R2L	995	0.790
Total	125,973	100.000

### Performance of the proposed IDS

In this subsection, the selection of a hyperparameter is conducted to find the best hyperparameter for the NSL-KDD dataset according to the hit rate value. From Table 12, it can be concluded that the lower value of *w* will produce a false alarm which can be seen from the high value of the FP rate. On the other hand, the larger value of *w* will reduce the ability of the proposed chart to detect the intrusion (higher FN rate). From the results, it can be concluded that *w* = 1 yields the higher Hit rate with balanced FP and FN rates. This also confirms the results of simulation studies in Section 3.3.

**Table 12:** Performance of proposed IDS for different hyperparameters.Significant values are in bold.

Hyperparameter (w)	Hit rate	AUC	FP rate	FN rate
0.10	0.9081	0.9088	0.1009	0.0815
0.25	0.9100	0.9097	0.0860	0.0946
0.50	0.9158	0.9146	0.0674	0.1035
0.70	0.9164	0.9150	0.0645	0.1056
1.00	**0.9171**	**0.9156**	**0.0625**	**0.1064**
1.50	0.9170	0.9155	0.0624	0.1065
2.00	0.9169	0.9154	0.0623	0.1069
2.50	0.9167	0.9152	0.0622	0.1074
3.00	0.9166	0.9151	0.0622	0.1077

### Comparison with the several IDS-based-control charts

This subsection elaborates the performance comparison of the proposed IDS based on the SVDD-KDE with several charts, such as Hotelling’s *T*^2^, and SDCM-based Hotelling’s *T*^2^ with several control limits as in (Ahsan et al., 2018). SDCM-F uses the *F* distribution control limit, SCDM-CH uses the Chi-square control limit, SDCM-SW uses the Sullivan and Woodall control limit^33^, and SDCM-MY uses the Mason and Young control limit^34^. Also, the SVDD-KDE chart is compared with Robuts Hotelling’s *T*^2^ chart with KDE, and Fast minimum covariance determinant (MCD) estimator (written as Fast MCD *T*^2^).

Table 13 tabulates the comparison of the proposed IDS based on SVDD-KDE with several IDS-based control charts. The results show that yields a similar Hit Rate SDCM-MY and Fast MCD *T*^2^. Compared to the SDCM-MY, the proposed IDS produces a smaller false alarm. Also, the proposed chart almost yields a similar result with IDS-based Fast MCD *T*^2^. Hence, it is deduced that the proposed SVDD-KDE chart has a higher accuracy and AUC in detecting intrusions with a lower false alarm. The drawbacks of the proposed SVDD-KDE is the high computational time.

**Table 13:** Performance comparison with several control charts.

Control charts	Hit rate	AUC	FP rate	FN rate	Computational time (in s)
*T*^2^	0.9133	0.9129	0.0937	0.0806	0.9197
SDCM-F	0.9134	0.9130	0.0937	0.0804	1.0152
SDCM-SW	**0.9171**	**0.9156**	0.1052	0.0636	1.0181
SDCM-MY	0.9133	0.9129	0.0937	0.0806	1.0167
SDCM-CH	0.9133	0.9129	0.0937	0.0806	1.0001
Fast MCD *T*^2^^23^	**0.9171**	**0.9156**	0.0624	0.1064	1.0789
Proposed chart (*w* = 1)	**0.9171**	**0.9156**	0.0625	0.1064	230.872

Significant values are in bold.

### Comparison with the several machine learning algorithms

This subsection discusses the performance comparison of the proposed chart with the other machine learning algorithms in monitoring the NSL-KDD dataset. The proposed IDS is compared with several machine learning algorithms such as the support vector machine, naïve Bayes, logistic regression, and decision tree. Based on the results in Table 14, it can be seen that the SVDD-KDE chart has the higher accuracy with the lowest false alarm rate (can be seen from the smaller value of the FP rate).

**Table 14:** Performance comparison with several machine learning algorithms.

Algorithms	Hit rate	FP rate
Hybrid naïve Bayes (NB)^8^	0.8239	0.1640
Naïve Bayes (NB)^2^	0.8729	0.1735
Logistic regression (LR)^4^	0.8400	0.1700
Support vector machine (SVM)^4^	0.7500	0.2400
Hybrid decision tree (DT)^8^	0.8192	0.1740
Proposed chart (*w* = 1)	**0.9171**	**0.0625**

Significant values are in bold.

## Conclusions

This research suggests a new multivariate chart based on the SVDD using KDE control limit, named the SVDD-KDE chart. From the simulations, the SVDD-KDE chart’s performance is inspected for monitoring process shifts, detecting outliers, and monitoring the simulated data. Further, the proposed IDS based on the SVDD-KDE chart is utilized to monitor the NSL-KDD dataset. Based on the simulation results, in detecting process shifts and outliers, the proposed chart performs better than the traditional *T*^2^ chart. When it is used to monitor the simulated dataset, the proposed chart demonstrates good results by correctly detecting the shift in the dataset. Furthermore, the proposed chart generates a higher Hit rate value with lower false alarms when it is applied to monitor attacks in the network. The limitation of this approach is the high time complexity. For future work, the Fast MCD estimator^23,24^ can be employed to enhance the detection rate of the proposed chart. The proposed chart have potential for large-scale attacks such as port scanning or distributed denial-of-service (DDoS.) and trojan or advanced persistent threat (APT) detection. Several methods such as one-class support vector machine (OCSVM), isolation forest (iForest), local outlier factor (LOF) as stated in^35^ can also be used to replace the SVDD method. Also, the bootstrap method^36^ can be utilized to compute the control limit of the IDS-base chart.

### Supplementary Information

Supplementary Information.

## Data Availability

The dataset is attached as a Supplementary File.
